# Investigating the Relevance of Graph Cut Parameter on Interactive and Automatic Cell Segmentation

**DOI:** 10.1155/2018/7396910

**Published:** 2018-09-13

**Authors:** Kazeem Oyeyemi Oyebode, Shengzhi Du, Barend Jacobus van Wyk, Karim Djouani

**Affiliations:** Department of Electrical Engineering, Tshwane University of Technology, Pretoria, South Africa

## Abstract

Graph cut segmentation provides a platform to analyze images through a global segmentation strategy, and as a result of this, it has gained a wider acceptability in many interactive and automatic segmentation fields of application, such as the medical field. The graph cut energy function has a parameter that is tuned to ensure that the output is neither oversegmented (shrink bias) nor undersegmented. Models have been proposed in literature towards the improvement of graph cut segmentation, in the context of interactive and automatic cell segmentation. Along this line of research, the graph cut parameter has been leveraged, while in some instances, it has been ignored. Therefore, in this work, the relevance of graph cut parameter on both interactive and automatic cell segmentation is investigated. Statistical analysis, based on F1 score, of three publicly available datasets of cells, suggests that the graph cut parameter plays a significant role in improving the segmentation accuracy of the interactive graph cut than the automatic graph cut.

## 1. Introduction

Graph cut segmentation technique has become popular in recent times because of its ability in segmenting images into foreground and background using a global strategy. Therefore, it has become a useful tool in many segmentation application areas. One of such areas is the medical field, where the application of graph cut yields promising results in cell [1] and lung [2] segmentation. The automatic graph cut segmentation is useful as it speeds up cell segmentation, while the interactive segmentation provides the flexibility to select seed points when further investigation needs to be carried out in isolation. An example is the segmentation of an infected cell, in a particular region of an image.

The graph cut energy function is equipped with a parameter (*λ*) which can be tuned to ensure that objects are not oversegmented and undersegmented. The graph cut parameter has been explored and exploited in the area of interactive segmentation with good results [3–5]. Candemir and Akgul [3] proposed a model where object boundaries are extracted and are used to adapt the graph cut parameter around object boundaries, their approach is similar to the use of shape prior to adapt segmentation around object boundaries in order to mitigate the shrinkage of the object size after segmentation [4, 5]. The graph cut parameter can also be selected based on some predefined quality attributes of object [6]. In addition, Kirmizigul and Schlesinger [7] proposed an interactive segmentation approach where a range of *λ* is considered, and when there is a significant difference in segmentation output within a considered range of *λ*, a further division is carried out until segmentation outputs are almost the same within a given *λ* range. This may be considered as a trial and error approach where *λ* is initialized with a value and which is constantly increased until further increments does not yield any improvement. A similar approach to Candemir and Akgul [3] is investigated where a canny edge detector is used to obtain object boundaries, which is used to influence how weights are assigned to graph edges in the graph context [8].

Another method of interactive segmentation is proposed [9] where the parameter is learnt from the image. First, the user draws a line along the boundary of object to be segmented, then the object is then stripped and its pixel properties, such as cohesiveness, are learnt and used to inform the graph cut segmentation. The proposed interactive approach has the advantage of being able to segment a single object. However, when multiple objects are required to be segmented, the interaction with each object's boundaries may be a tedious task to undertake. The selection of *λ* based on experimental values, for cell segmentation, has also been researched [1]. A learning process for graph cut parameter is proposed [10] where segmentation is carried out iteratively. After each iteration, the segmentation result is compared with the ground truth, and then the graph cut parameters are adjusted in the next iteration to reflect an improved segmentation output over that obtained in the previous iteration. This is done until the recent segmentation output and the ground truth are almost similar. This approach to parameter learning may not be useful when ground truth of images is not available. Other related works [11–14] in respect of the selection of an appropriate parameter for image restoration have also been discussed. In addition, other approaches such as the Otsu thresholding, the k-means, and the template matching algorithms [15] have also been explored for cell segmentation. While some of the interactive segmentation methods proposed adapted *λ* in their graph cut methods, many automatic graph cut segmentation processes are carried out while ignoring the *λ* [16–18].

The focus of this paper is in three folds. Firstly, the relevance or the usefulness of graph cut parameter on graph cut segmentation is investigated. Admittedly, some existing researches have focused on investigating an optimal approach to graph cut parameter selection as discussed earlier. Secondly, the question of whether the graph cut parameter is useful to the investigation of both interactive and automatic segmentation is considered. This is a crucial consideration since most of the existing parameter selections focus on interactive segmentation only. Thirdly, the investigation of the effect of noise, on both interactive and automatic cell segmentation is carried out with respect to a constant *λ*. To the best of our knowledge, the investigation of the relevance of the graph cut parameter, in interactive and automatic cell segmentation, has not been carried out before.

## 2. Materials and Methods

### 2.1. A Graph

**Figure 1:**
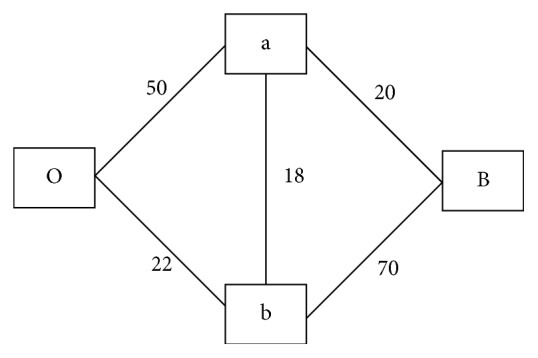
Weighted graph.

A graph *G* = (*V*, *E*), can be interpreted as having a set of nodes *V* and set of edges *E*. An example of this kind of graph is shown in Figure 1. In Figure 1, a, b, O, and B are nodes while O−a, a−B, b−B, a−b, and O−b are edges with corresponding weights 50, 20, 70, 18, and 22.

The idea behind the graph cut method is to discover, within a graphical network, the edge with the least flow capacity (edge with maximum flow, since the least capacity edge will have the maximum flow). A simple way to achieve this is to increase the flow (in this case liquid) from source node O to B (Figure 1). An edge capacity in the network may reach its saturation point, thereby be unable to accommodate further increase in the flow of liquid from O to B. At this point, the weakest link has been found in the network.

### 2.2. Graph Cut Segmentation

The objective of graph cut segmentation is to assign a label *S ϵ* {0, 1} to each pixel in a given image *I* where label “1” represents the foreground and “0” represents the background. Given *I* with observed grey-scale intensity level *M ϵ* {*M*_o_, *M*_b_} (where *M*_o_ and *M*_b_ are observed foreground and background intensity levels), with *x* number of pixels, then the segmentation (*S*) of *I* into foreground and background, using the Bayesian model, is formulated in Equation (1), and *I*_*a*_ is the grey-scale intensity level of pixel *a*:

(1) $$\begin{array}{c} P\left(S\;|\;M\right)=\left(\overset{x}{\underset{a=1}{\prod }}P\left(I_{a}\;|\;S\right)\;*\;P\left(S\right)\right). \end{array}$$

The maximum a posteriori (MAP) estimation for the segmentation of *I* is given in the following equation:

(2) $$\begin{array}{c} S_{\text{MAP}}=\arg\max_{k}\left(\overset{x}{\underset{a=1}{\prod }}P\left(I_{a}\;|\;S\right)\;*\;P\left(S\right)\right). \end{array}$$

The negative logarithm of MAP in Equation (2) gives the following equation:

(3) $$\begin{array}{c} E\left(S\right)-\log\left(\overset{x}{\underset{a=1}{\prod }}P\left(I_{a}\;|\;S\right)\right)-\log\;P\left(S\right), \end{array}$$

where *E*(*S*) is the energy function that needs to be minimized in order to partition *I* into foreground and background. *E*(*S*) can also be rewritten as seen in the following equation:

(4) $$\begin{array}{c} E\left(S\right)-\log\left(\overset{x}{\underset{a=1}{\prod }}-\log\left(P\left(I_{a}\;|\;S\right)\right)\right)-\log\;P\left(S\right). \end{array}$$

In Equation (4), −log *P*(*S*) can be represented as a Markov Random Field (MRF) pairwise interaction between neighbouring pixels [19] *a* and *b* in Equation (5) where *σ* describes pixel similarity and *N* encapsulates neighbourhood pixels.

(5) $$\begin{array}{c} -\log\;P\left(S\right)=\underset{\left(a,b\right)\in N}{\sum }\exp\left(-\frac{I_{a}-I_{b}}{2\sigma^{2}}\right). \end{array}$$

Therefore, the energy function can be rewritten as seen in the following equation:

(6) $$\begin{array}{c} E\left(S\right)=\lambda \left(\overset{x}{\underset{a=1}{\sum }}-\log\left(P\left(I_{a}\;|\;S\right)\right)\right)+\underset{\left(a,b\right)\in N}{\sum }\exp\left(-\frac{{\left|I_{a}-I_{b}\right|}^{2}}{2\sigma^{2}}\right). \end{array}$$

In Equation (6), the first part of the equation is referred to as the data term while the second part is called the smoothness term. The parameter *λ* adjusts the relative importance of the data term to the smoothness term. There are several algorithms that can be used to minimize the energy function in Equation (6). One of such is the Ford Fulkerson algorithm [20]. Other algorithms [19, 21] are also proposed.

The Ford–Fulkerson [20] algorithm partitions a graph into two parts that are disjoint. In the image context, the image is partitioned into foreground (O) and background (B). The algorithm does this by finding the weakest link in a weighted graph network *G* of Figure 1. The weakest link(s) found globally (along the entire graph) invariably partition(s) the image into foreground and background. When this occurs, the algorithm has found the minimum cut (weakest link), where the maximum flow occurs. Assuming the data term in Equation (6) is used to assign weights to edges O–a, B–a, O–b, and B–b and the smoothness term is used to assign weight to the edge a–b in Figure 1, then Ford Fulkerson algorithm can be used to partition the graph into foreground (O) and background (B) as follows:

1. Find the unsaturated path linking nodes O and B
2. Saturate the discovered path with the minimum edge capacity in step 1
3. Repeat steps 1 and 2 until all, path linking nodes O and B, are saturated

### 2.3. Investigating the Relevance of Graph Cut Parameter on Interactive and Automatic Cell Segmentation

The graph cut parameter within the context of the interactive and automatic segmentation on homogeneous, fairly homogeneous, and heterogeneous cell images is investigated. In both interactive and automatic cell segmentation strategies, the adaptation of the graph cut parameter is carried out at the cell boundaries in order to find out its relevance in mitigating the reduction in the size of objects (shrink bias). Shrink bias occurs when the boundary pixels of an object are absent after segmentation. It results in cells losing their actual size.

The approach of adapting the graph cut parameter, through object boundaries, is inspired by models discussed earlier [3, 4, 8], where the objective is to mitigate the shrink bias of graph cut. However, cell boundaries are extracted as discussed in [22]. Furthermore, the graph cut parameter value is varied to investigate its impact on the interactive and automatic graph cut segmentation. This approach is also similar to the model proposed in [7]. Equation (8) is used to adapt *λ* in Equation (7), while *a*_E_ is the set encapsulating boundary pixels (Equation (9)). Equation (9) shows how *c* is manipulated to adapt *λ* in Equation (8). In Equation (6), *λ* is set to 20, also in Equation (8), *λ*_1_ is set to 20. An initial value of 20 is selected to ensure the graph cut parameter is not too large nor not too small. In Equation (9), *c*_p_ is also set to 20.

(7) $$\begin{array}{c} E\left(S\right)=\lambda \left(\overset{x}{\underset{a=1}{\sum }}-\log\left(P\left(I_{a}\;|\;S\right)\right)\right)+\underset{\left(a=1\right)\in N}{\sum }\exp\left(-\frac{\left|I_{a}-I_{b}\right|}{2\sigma^{2}}\right), \end{array}$$

(8) $$\begin{array}{c} \lambda =\lambda_{1}\;*\;c, \end{array}$$

(9) $$\begin{array}{c} c=\left\{\begin{array}{cc} c_{\text{p}}, & a\;\epsilon \;a_{\text{E}}\text{ at edge}\left(a–0\right),\\ 0, & a\;\epsilon \;a_{\text{E}}\text{ at edge}\left(a–B\right),\\ 1, & a\;\notin \;a_{\text{E}}. \end{array}\right. \end{array}$$

**Figure 2:**
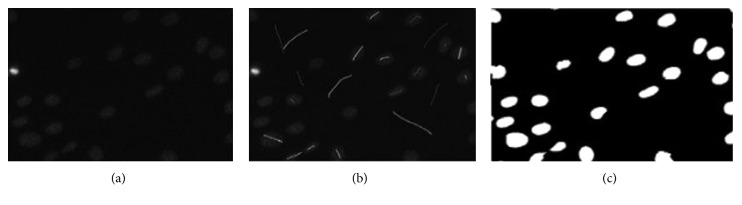
(a) Cell image. (b) Manual selection of sample foreground and background pixels. (c) Automatic selection of sample foreground and background pixels via Otsu thresholding.

The interactive segmentation provides a suitable platform to select foreground and background seed points on cell images. These seed points represent the observed intensity level *M*_O_ for foreground and *M*_B_ for background.  Figure 2(b) shows how *M*_O_ and *M*_B_ are selected interactively. In addition, Figure 2(c) shows how *M*_O_ and *M*_B_ are selected automatically from the Otsu segmentation (white represents *M*_O_ and black represents *M*_B_). *M*_O_ and *M*_B_ are used to build histograms of pixel intensity distribution for both foreground and background. These histograms are used to calculate the negative logarithm of the probability (data term in Equation (7)) of a given pixel intensity *I*_*a*_ being foreground (a–O) and background (a–B).

In the interactive approach, two types of interactive cell segmentation techniques are proposed. The first approach segments cell images with the static graph cut parameter (as observed in Algorithm 1), while the second segments with the adaptive graph cut parameter (Algorithm 2). As regards adapting *λ* on cell segmentation (Equation (7)), boundaries of cells are extracted as discussed in [22].

In the automatic cell segmentation, sample foreground and background pixels are selected automatically (Figure 2(c)). The selection is carried out on an Otsu segmented image to provide a coarse initial segmentation which serves as input for the selection of sample foreground and background pixels (seed points). This process is done automatically. The extraction of cell boundaries for the adaptation of graph cut parameter value is also undertaken as observed in [22]. This development gives rise to two kinds of automatic cell segmentation—the graph cut parameter when static *λ* (Algorithm 1) and the automatic cell segmentation (Algorithm 2) while adapting the graph cut parameter. In the evaluation section, the effect of noise on a given *λ* is also investigated.

## 3. Evaluation

The segmentation accuracies of the models are evaluated using the Accuracy Index (AI) metric (Equation (10)) and the F1scoremetric (Equation (11)). High values of AI and F1 score give good segmentation result. The F1 metric is also leveraged to investigate the statistical significance of a given model over another. The effect of noise is investigated on both interactive and automatic segmentation given a constant *λ*. The graph cut parameter is also varied to analyze its impact on the interactive and automatic segmentation. Lastly, segmentation accuracies of models are also investigated under the Receiver Operating Characteristic (ROC) curves. The ROC curves give an account of the segmentation performance of a model using its false negative rate against its true positive rate. The Area Under the Curve (AUC) of a given ROC is then observed to determine its performance. An AUC close to 1 gives good segmentation output.

The AI metric evaluates segmentation accuracies based on the total number of correctly labeled pixels; it does not give an account of how a model performs based on its precision and recall, this is where the F1 metric becomes useful (Equation (11)), and it gives an account of how a model performs using the recall and precision. The ROC curves also investigate the performance of a model leveraging on its true positive and true negative rates.

**Figure 3:**
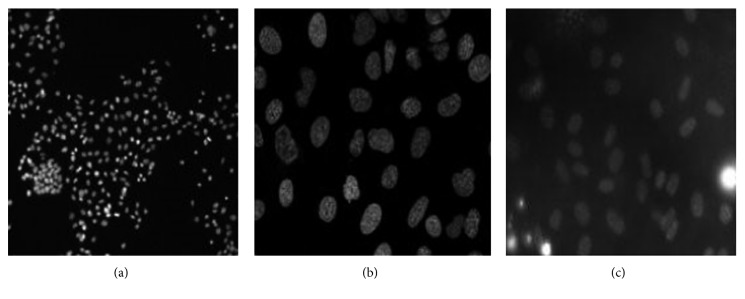
Sample dataset. (a) HT29. (b) U2OS. (c) NIH3T3.

Three publicly available datasets have been used for evaluation. The first is the U2OS [15] (1831 of fairly homogeneous cells of 49 images). The second is NIH3T3 [15] (2178 of heterogeneous cells of 49 images) while the third is the HT29 [23] (1291 of homogeneous cells of 24 images). These datasets are accompanied with their corresponding ground truths. Sample images of these datasets are shown in Figure 3. The graph cut algorithm proposed by Boykov and Jolly [21] is leveraged for the experiment, and its MATLAB implementation can be found in [24].

(10) $$\begin{array}{c} \text{AI}=\frac{\text{TP}+\text{TN}}{\text{TP}+\text{TN}+\text{FP}+\text{FN}}, \end{array}$$

(11) $$\begin{array}{c} \text{F}1\text{ score}=2\cdot \left(\frac{\text{precision }*\text{ recall}}{\text{precision}+\text{recall}}\right), \end{array}$$

(12) $$\begin{array}{c} \text{precision}=\left(\frac{\text{TP}}{\text{TP}+\text{FP}}\right), \end{array}$$

(13) $$\begin{array}{c} \text{recall}=\left(\frac{\text{TP}}{\text{TP}+\text{FN}}\right). \end{array}$$

True positive (TP) is the total number of foreground pixels found in the segmented image S (binary) that are found to be foreground pixels in the gold standard (ground truth) *G*. True negative (TN) is the total number of background pixels in the segmented image S that are found to be background pixels in *G*. False positive (FP) is the total number of foreground pixels in the segmented image S that are found to be background pixels in *G*. False negative (FN) is the total number of background pixels in the segmented image S that are found to be foreground pixels in *G*.

## 4. Results

### 4.1. Investigating the Relevance of Graph Cut Parameter on Interactive and Automatic Cell Segmentation

In Table 1 (where std is standard deviation), the segmentation results obtained by using the interactive graph cut segmentation is shown. It depicts that *λ* is both static and adaptive. On the U2OS dataset, it can be observed that the value of F1 (interactive segmentation) when *λ* is adaptive is high compared to when *λ* is static. This indicates that the shrink bias (reduction in the actual size) of graph cut is minimized when the graph cut parameter is adaptive. It can also be observed in Table 1, that is when *λ* is adaptive, a value for FN gives a score of 51947, whereas a score of 92152 is recorded when *λ* is static. This trend can also be observed in Tables 2 and 3. However, in Tables 4–6, one would notice that the F1 values are approximately the same when compared to the values of F1 in Tables 1–3.

In Tables 1–3, a reduction in the shrink bias of graph cut is observed (FN metric). There is a significant difference between the values of FN in the referenced tables. This is because the sample foreground pixels selected by the user (*M*_O_) may not cover, sufficiently, the intensity levels of all foreground pixels in an image (including foreground boundary pixels). Hence, the introduction of adaptive *λ* helps to increase the edge weight (a–O) of pixels around cell boundaries and therefore reduces the graph cut shrink bias. The absence of this may result in cells losing their boundaries (after segmentation), culminating in the high FN value when *λ* is static (Tables 1–3). However, in Tables 4–6, the selection of foreground and background sample pixels are carried out automatically on an initial Otsu segmented image. This ensures that the variability of intensity levels of foreground pixels (*M*_O_) is sufficiently captured. Thus, the assignment of edge weight reflects the true intensity level of pixels. As a result, adapting *λ* may have minimal effect on the shrink bias of graph cut as observed in F1 values in Tables 4–6. This analysis also applies to the AI index in all the six tables.

**Figure 4:**
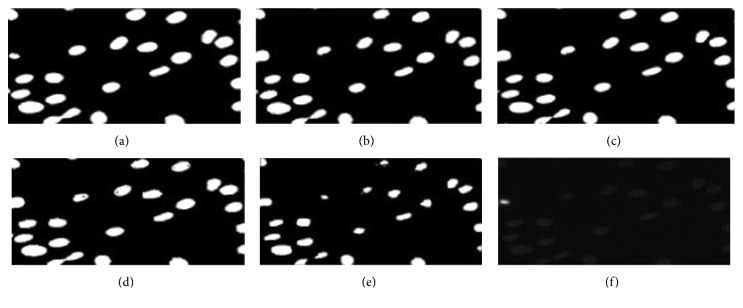
(a) Ground truth. (b) Automatic segmentation with adaptive *λ*. (c) Automatic segmentation with static *λ*. (d) Interactive segmentation with adaptive *λ*. (e) Interactive segmentation with static *λ*. (f) Original image.

Figure 4 reinforces the argument, of the shrink bias, put forward. In Figure 4(b), an automatic segmentation of cells (Figure 4(f)) with adaptive *λ* is seen while in Figure 4(c), it is static. One can barely spot the differences in cell sizes in the two images. However, in Figures 4(d) and 4(e), there is a clear difference in cell sizes. The cells in Figure 4(d) appear bigger than that in Figure 4(e). It is obvious that cell boundaries are omitted in Figure 4(e) owing to the shrink bias of graph cut.

### 4.2. Statistical Significance Test of Accuracy

In order to investigate the significance of the difference in the accuracy of the interactive graph cut segmentation over the automatic graph cut model, a *t*-test is carried out on the F1 metric. The F1 metric is considered as it combines the precision and recall of any segmentation output. The *t*-test is a statistical test which indicates whether there exists a statistical significance in the segmentation accuracy of a given model over another using the F1 metric. If a *p* value obtained from the *t*-test > 0.05 [25], then there is no statistical significance in F1 metric between two models. However, if the *t*-test < 0.05, then there exists a statistical significance. Equation (14) gives the *t*-test formula:

(14) $$\begin{array}{c} t‐\text{test}=\frac{M_{2}-M_{1}}{\sqrt{\left({\text{SD}}_{2}/N\right)-{\left({\text{SD}}_{1}/N\right)}^{2}}}. \end{array}$$

In Equation (14), *M*_2_ and *M*_1_ give the mean values of F1 score, *N* is the number of cell images in the considered dataset, and SD_2_ and SD_1_ are standard deviations of models in a considered table.

Table 7 shows the statistical significance of adapting graph cut parameter over the interactive and automatic segmentation. The interactive segmentation of cells when *λ* is adaptive shows statistical significance over when *λ* is static. Hence, the contribution of adaptive *λ* on interactive cell segmentation is significant in all the three datasets. However, there is no statistical significance over the automatic segmentation.

### 4.3. Varying Graph Cut Parameter on the Interactive and Automatic Segmentation

**Figure 5:**
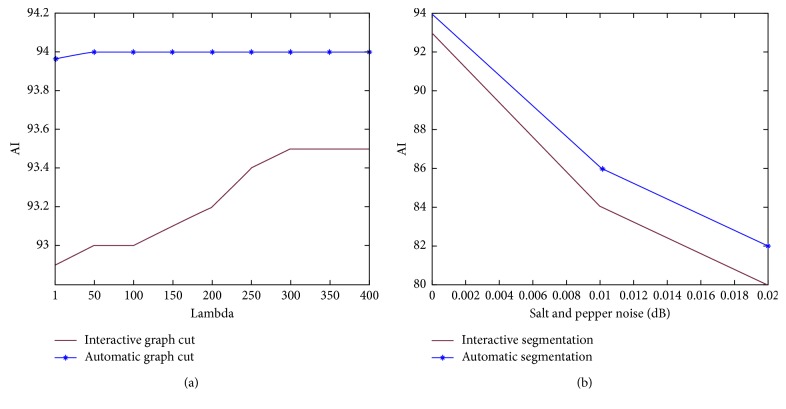
(a) Segmentation accuracies when *λ* is varied on the U2OS dataset. (b) Given a constant *λ* of 20, segmentation accuracy decreases with increase in noise intensity on the U2OS dataset.

As observed in Figure 5(a), different segmentation accuracies are observed with different values of *λ* (1 to 400). This development shows that varying the graph cut parameter may influence segmentation output, confirming the claim in [21]. However, the significance of varying *λ* on automatic segmentation is negligible. One explanation to this is that the variability of the grey-scale intensity levels of foreground pixels is sufficiently captured by the automatic selection of seed points. Hence, varying *λ* in order to add weights to graph edges may not be necessary. However, for interactive segmentation, *λ* may influence its segmentation output as its interactive method of seed selection may not have covered sufficiently the variability of foreground intensity levels.

### 4.4. Lambda (*λ*) Performance on Noisy Cell Images

As observed in Figure 5(b), the increase in the intensity of “salt and pepper” noise, given that *λ* has a constant value of 20, has a negative effect on the segmentation output on both interactive and automatic segmentation.

### 4.5. Receiver Operating Characteristic (ROC) Curves

**Figure 6:**
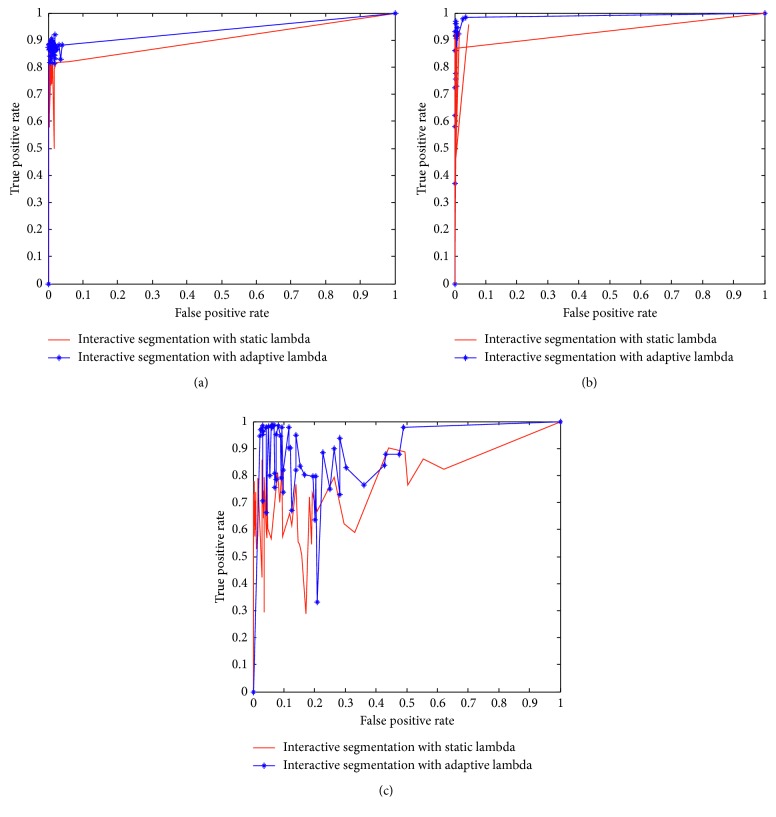
ROC curves for the interactive segmentation when *λ* is static and adaptive on the (a) U2OS dataset. (b) HT29 dataset. (c) NIH3T3 dataset.

Figure 6 shows the Receiver Operating Characteristic (ROC) curves for the three datasets (interactive segmentation).  Table 8 also shows the Area under Curve (AUC) for the ROC curves. The AUC close to 1 suggests good segmentation result.

Table 9 compares the best segmentation outputs from Tables 1–6 to existing segmentation models. The Otsu thresholding which is used to autoselect seed points for the automatic segmentation has segmentation outputs of 92/74/89 on U2OS, NIH3T3, and HT29 datasets, respectively. The merging algorithm has 96 % segmentation accuracy on the U2OS dataset; hence, it outperforms the best result of 95.3 % obtained from Tables 1–6.

## 5. Discussion

The outcome of the investigation, carried out on the three publicly available datasets, suggests that the graph cut parameter (*λ*) plays a significant role in improving the segmentation accuracy and the reduction of graph cut shrink bias on interactive cell segmentation. However, its impact on automatic segmentation is negligible. Where appropriate tools have been deployed with a view to enhancing the output of automatic graph cut segmentation, the accuracy of automatic graph cut segmentation may not be significantly affected where *λ* is ignored. Thus, *λ* plays a significant role in interactive graph cut segmentation, although the performance of both (interactive and automatic segmentation) could be adversely affected by cell-image noise. Automatic graph cut segmentation is useful as it speeds up cell segmentation. However, when an area of an image is subjected to further investigation, in isolation, then the interactive segmentation has its own advantage because it enables seed points to be selected interactively.

The automatic graph cut segmentation outperforms the interactive segmentation for one reason. As can be observed in Figures 2(b) and 2(c), the automatic segmentation captures the variability of foreground intensity levels better than the interactive segmentation.

## 6. Conclusion

This paper has investigated the relevance of the graph cut parameter (*λ*) in interactive and automatic graph cut cell segmentation strategies (using more than 5000 cells). Based on the investigation performed, this establishes three novel conclusions: (1) the adaptation of the graph cut parameter across various regions of the cell image minimizes the shrink bias of the interactive graph cut segmentation; (2) the adaptation of the graph cut parameter value may significantly improve segmentation performance for the interactive graph cut than the automatic graph cut; and (3) the presence of noise on cell images may reduce the performance of a chosen graph cut parameter value.

## Figures and Tables

**Algorithm 1:**
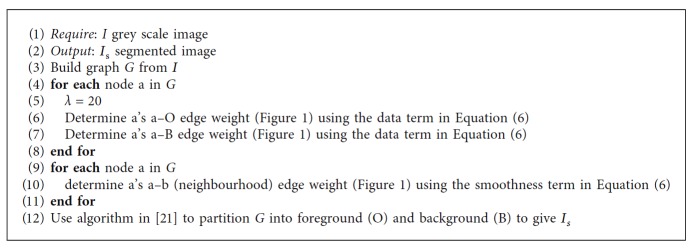
Cell segmentation using Equation (6).

**Algorithm 2:**
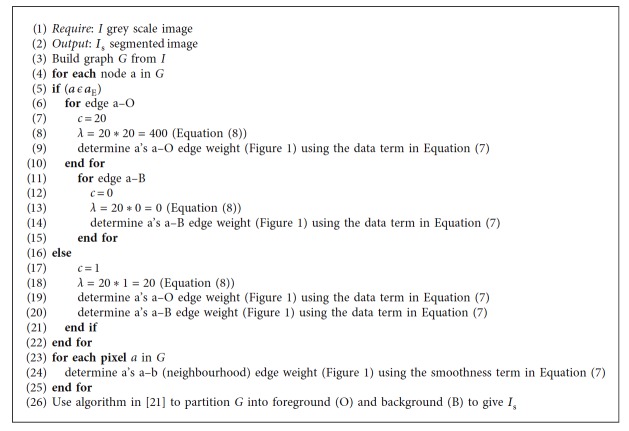
Cell Segmentation using Equation (7).

**Table 1:** Interactive graph cut segmentation using the U2OS dataset.

Model	AI (%)	F1 (±std)	FN	FP	TP	TN
Interactive (*λ* static)	92.9	86 ± 4	92152	6779	302411	981087
Interactive (*λ* adaptive)	95.30	93.2 ± 2	51947	13132	340122	977925

**Table 2:** Interactive graph cut segmentation using the NIH3T3 dataset.

Model	AI (%)	F1 (±std)	FN	FP	TP	TN
Interactive (*λ* static)	82.9	65.5 ± 15	99621	134586	204291	937757
Interactive (*λ* adaptive)	85.4	73.8 ± 15	43497	156705	260414	915638

**Table 3:** Interactive graph cut segmentation using the HT29 dataset.

Model	AI (%)	F1 (±std)	FN	FP	TP	TN
Interactive (*λ;* static)	93.5	77 ± 18	16300	735	29335	215772
Interactive (*λ* adaptive)	95.46	86 ± 9	10566	1331	36496	213750

**Table 4:** Automatic graph cut segmentation using the U2OS dataset.

Model	AI (%)	F1 (±std)	FN	FP	TP	TN
Automatic (*λ* static)	93.96	89 ± 3	75452	7584	321519	976087
Automatic (*λ* adaptive)	94	88.2 ± 4	75052	7601	315401	978199

**Table 5:** Automatic graph cut segmentation using the NIH3T3 dataset.

Model	AI (%)	F1 (±std)	FN	FP	TP	TN
Automatic (*λ* static)	85.3	70 ± 13	88923	113328	214989	959015
Automatic (*λ* adaptive)	85.8	70 ± 14	76778	117835	227133	954508

**Table 6:** Automatic graph cut segmentation using the HT29 dataset.

Model	AI (%)	F1 (±std)	FN	FP	TP	TN
Automatic (*λ* static)	96	88 ± 1	3019	7419	40043	212428
Automatic (*λ* adaptive)	96	88 ± 1	3019	7420	40043	212430

**Table 7:** Statistical significance test.

Model	*T*-test	*p* value	Statistical significance F1 score
Interactive (*λ* adaptive and static) U20S	11.25	0.01	86 is statistically significant over 93.2
Automatic (*λ* adaptive and static) U20S	1.11	0.2	89 is not statistically significant over 88.2
Interactive (*λ* adaptive and static) NIH3T3	2.7	0.01	65.5 is statistically significant over 73.8
Automatic (*λ* adaptive and static) NIH3T3	0	0.2	70 is not statistically significant over 70
Interactive (*λ* adaptive and static) HT29	2.1	0.02	77 is statistically significant over 86
Automatic (*λ* adaptive and static) HT29	0	0.2	88 is not statistically significant over 88

**Table 8:** Automatic graph cut segmentation using the U2OS dataset.

Dataset	Area under curve
U2OS interactive (*λ* static)	0.95
U2OS interactive (*λ* adaptive)	0.96
HT29 interactive (*λ* static)	0.97
HT29 interactive (*λ* adaptive)	0.98
NIH3T3 interactive (*λ* static)	0.74
NIH3T3 interactive (*λ* adaptive)	0.84

**Table 9:** Comparison of segmentation models.

Model	AI % (U2OS/NIH3T3/HT29)
Otsu thresholding [15]	92/74/89
Watershed [15]	91/78/-
Merging algorithm [15]	**96**/83/-
K-means	92.4/83/89.3
Best results from Tables 1–6	95.3/**85.8/96**

## Data Availability

The cell image datasets NIH3T3 and U2OS have been referenced in [15]. In addition, these datasets can be downloaded from http://murphylab.web.cmu.edu/data/. The cell image dataset HT29 has been referenced in [23]. In addition, these datasets can be downloaded from https://data.broadinstitute.org/bbbc/BBBC008/ or from the corresponding author upon request.
